# Dealing with taste and smell alterations—A qualitative interview study of people treated for lung cancer

**DOI:** 10.1371/journal.pone.0191117

**Published:** 2018-01-23

**Authors:** Kerstin Belqaid, Carol Tishelman, Ylva Orrevall, Eva Månsson-Brahme, Britt-Marie Bernhardson

**Affiliations:** 1 Medical Management Centre, Department of Learning, Informatics, Management and Ethics, Karolinska Institutet, Stockholm, Sweden; 2 Function Area Clinical Nutrition, Karolinska University Hospital, Stockholm, Sweden; 3 Centre for Innovation, Karolinska University Hospital, Stockholm, Sweden; 4 Cancer Theme, Karolinska University Hospital, Stockholm, Sweden; 5 Department of Oncology-Pathology, Karolinska Institutet, Stockholm, Sweden; University of Liverpool, UNITED KINGDOM

## Abstract

Taste and smell alterations have been recognized as common symptoms in relation to various cancers. However, previous research suggests that patients do not receive sufficient support in managing taste and smell alterations. Therefore, the objective of this study is to investigate how persons with experience from lung cancer-related taste and smell alterations reason about resources and strategies offered and used to manage these symptoms. Data from semi-structured individual interviews with 13 women and four men were analyzed with qualitative content analysis. We used Kleinman’s now classic medical anthropological model of local health care systems, consisting of the personal, professional, and folk sector, to interpret and understand how people respond to sickness experiences in their daily lives. By presenting the findings using this model, we demonstrate that most strategies for dealing with taste and smell alterations were undertaken in the personal sector, i.e. in participants’ daily lives, on an individual level and in interaction with family, social networks and communities. Taste and smell alterations implied two overarching challenges: 1) adjusting to no longer being able to trust information provided by one’s own senses of taste and/or smell, and 2) coming to terms with taste and smell alterations as a part of having lung cancer. Health care professionals’ involvement was described as limited, but appeared to fulfil most participants’ expectations. However, through provision of normalizing information, practical advice, and to some extent, emotional support, health care professionals had potential to influence strategies and resources used for dealing with taste and smell alterations. With this study, we further the understanding of how people deal with lung cancer-related taste and smell alterations and discuss the role of health care professionals for this process.

## Introduction and aim

Taste and smell alterations (TSAs) have been recognized as a common problem for people with a variety of cancers. Although they have primarily been studied as side-effects of treatments such as chemotherapy and radiotherapy [1, 2], there is also evidence suggesting that TSAs may present in palliative disease phases [3], in relation to targeted therapy [4], and among people with lung cancer even before treatment start [5, 6]. Characteristics of TSAs may include increased or decreased sensitivity in one or more of the five basic taste qualities (sweet, sour, salt, bitter, umami) or smell, as well as altered sensory experiences of specific tastes, foods, or odors [7–9]. These characteristics may vary both between individuals and for the same individual over time [10]. Although chemotherapy-related TSAs have been reported to present early in a chemotherapy cycle and thereafter come and go intermittingly in relation to treatment administration [7], there are also reports of TSAs being constant through chemotherapy treatment [11]. Furthermore, other eating related symptoms, e.g. nausea, oral problems, early satiety and loss of appetite have been found to be interrelated with TSAs [3, 9, 11, 12]. Several authors report that TSAs alter food enjoyment, preferences and intake, thereby potentially contributing to involuntary weight loss and undernutrition [3, 13]. Persons with cancer-related TSAs may also perceive them as causing negative emotions such as disappointment, frustration and sadness, interfering with social rituals around meal situations, and altering routines and roles within the family [12, 14, 15].

While there is a lack of documented effective treatments for alleviating TSAs [16], a few studies report how people find ways to avoid unpleasant taste or smell experiences, or to manage social consequences of TSAs [17, 18]. Although health care professionals have been reported to provide information about TSAs as possible side-effects before or during chemotherapy, the described strategies were generally developed by those experiencing TSAs themselves without much support from health care professionals [14, 17]. In addition, some authors suggest that TSAs may be overlooked by health care professionals [12, 19, 20]. Proposed reasons for this include underreporting of TSAs by patients [14, 15, 17] and a lack of bio-medically-based professional solutions to alleviate TSAs or restore taste or smell function [12]. Oncological clinicians’ self-perceived difficulties supporting people with TSAs were discussed in an Australian study of dietitians’, nurses’ and physicians’ experiences [19]. These clinicians talked about a need for tools for assessment and diagnosis of taste changes, as well as evidence-based treatment strategies, able to be tailored to individual TSA characteristics, to improve their practice.

The importance of developing evidence-based guidelines for management of TSAs has been emphasized in several research reports [1, 16, 21]. Common suggestions put forth for further research to inform this process include investigation of biological mechanisms, systematic evaluations of TSAs’ characteristics and consequences, and clinical trials to evaluate effectiveness of interventions. Without discrediting the value of generating this kind of knowledge, we argue that given the reported individuality and variety in TSA characteristics and consequences, an additional and important type of complementary information would be how people with experience from TSAs themselves deal with TSAs when they occur. Such insights could identify prioritized areas for improvement of TSA management. Therefore, the aim of this qualitative study is to investigate how people with experience of cancer-related TSAs reason about the resources and strategies for dealing with these symptoms that may have been used and/or offered, including support from health care professionals and other sources.

## Methods

This study was conducted within the Swedish ‘Taste and Smell project’ approved by the Regional Ethical Review Board (2009/1463-31/3; 2010/1849-32; 2011/1324-32, 2014/154-31). To complement previously collected structured and primarily quantifiable data on TSAs among people with lung cancer (see e.g. [9, 10] for further description), we performed 17 qualitative interviews with persons with lung cancer and experience of TSAs who had not otherwise participated in the project and were unknown to the researchers. This study was inspired by interpretive description, a qualitative approach for generating knowledge about clinical problems relating to human experiences through description and interpretation of patterns of experience, action, or expression [22]. As this approach is rather generic, the specific procedures used are described in detail below.

### Recruitment of participants

Purposeful recruitment of participants was conducted from April to June 2014 and September 2015 to June 2016. As often the case in qualitative research, the exact number of participants was not decided on in advance but estimated to between 15–20 participants, based on the richness of the interview data. Eligible persons were those with a diagnosis of lung cancer and experience of self-defined taste and/or smell alterations which were first noted in relation to the disease or its treatment. We employed two different strategies for recruitment: 1) Sixteen patients with known TSAs were first approached by dietitians or nurses at the two sites of the lung medicine department at one university hospital in an urban area in Sweden. If interested in participation they were contacted by the first author. Of the four approached patients who declined to participate, three provided explanations for their decision: one lacked energy for study participation, one had a sudden injury, and one did not have time. 2) Information about the study was also distributed via the Swedish Lung Cancer Advocacy organization’s internet home page. Of the seven interested persons who contacted the first author by e-mail or telephone, two were found to be ineligible for the study, one was not possible to later reach, and four were interviewed. One additional participant was recruited by direct contact through the advocacy organization.

### Data collection and analysis

After providing written and verbal information about the project and obtaining written informed consent, semi-structured individual interviews took place at a location of the participants’ choice. Fifteen interviews took place at the participant’s home, one at an outpatient ward, and one was conducted by telephone. The interviews lasted 20–100 minutes, with a median of about 50 minutes. Participants were informed that this study involved a single interview, although the interviewer concluded the interview by asking for permission to contact the participant if further questions arose and invited the participant to contact the interviewer at any time. Only one participant later contacted the interviewer, to provide complementary information on dietary changes she had made some months before the interview, based on media reports of cancer preventive diets. Participants did not review interview transcripts. All interviews were conducted in conversational form by the first author (KB) and supported by an interview guide. KB is a female registered dietitian and PhD student within the Taste and Smell project, trained in qualitative research and supervised by a multi-professional team of experienced health care researchers with clinical experience of working with this patient group. None of the researchers, including KB, had any pre-existing or professional relationship with the participants.

Field notes were written after each contact with participants, including detailed field notes immediately after interviews. All interviews were audio-recorded and later verbatim transcribed by a professional transcriber. After receiving each interview transcript, KB undertook an initial reading while listening to the audio-tape and correcting the transcript. This process entailed being sensitive for preliminary patterns in the data, which were documented in reflective memos, and informed following interviews.

Interviews were carried out in conversational form and were initiated with the following open-ended question by the interviewer: *Could you please tell me about any changes you’ve been experiencing with your senses of taste and smell changes at the moment*? Follow-up questions, in line with the interview guide, were based on the participants’ descriptions and responses. The initial interview guide was developed by the research team, to address existing knowledge gaps and had three main topics: 1) participant’s experiences of TSAs (characteristics, timing, impact on daily life, and how bothersome they were); 2) how participants made efforts to manage TSAs including their rationale and perceived helpfulness of these strategies, and 3) information about and support for TSAs (content, timing, and sources of information, with whom participants’ had discussed TSAs, and advice and support offered). The interview guide was updated twice, after having completed four and nine interviews, respectively, to enable better probing of what we initially saw as the participants’ often rather limited descriptions about provision of information about and support for TSAs. The revisions of the interview guide included probes about thoughts and feelings in situations where TSAs interfered with daily life, questions about importance and meaning of food and mealtimes for the participant, and how TSAs may have interacted with other issues or symptoms that had arisen during the sickness trajectory.

During a pause in recruitment after completing nine interviews, KB reread these interviews and began formal inductive coding using the qualitative analysis software NVivo 11. Coding was performed by KB, with support from the last author (BMB), an experienced researcher in qualitative methodology and TSAs. Two interviews were coded by KB and BMB together; thereafter KB coded the data independently but with regular support from BMB regarding the codes and the coding procedures. Initial codes remained close to the data, and were subsequently brought together into broader categories, corresponding to the study aim. When recruitment was resumed, coding was undertaken after each interview. During this process, codes were continually adjusted into six larger, but still data-derived, descriptive categories based on regular discussions between KB and BMB. These categories were *Descriptions of TSAs*, *Consequences of TSAs*, *Attitudes to TSAs*, *Behaviors and strategies for dealing with TSAs*, *Advice and support for TSAs*, and *Circumstances*, with the latter including data on e.g. the lung cancer diagnosis, other symptoms, and relationships to food and mealtimes, as contextualizing findings is an important aspect of qualitative research [23]. The analysis was discussed within the full research team on several occasions during this process, with particular focus on contextualizing the strategies and resources participants used for dealing with TSAs and the described role of health care professionals.

**Fig 1:**
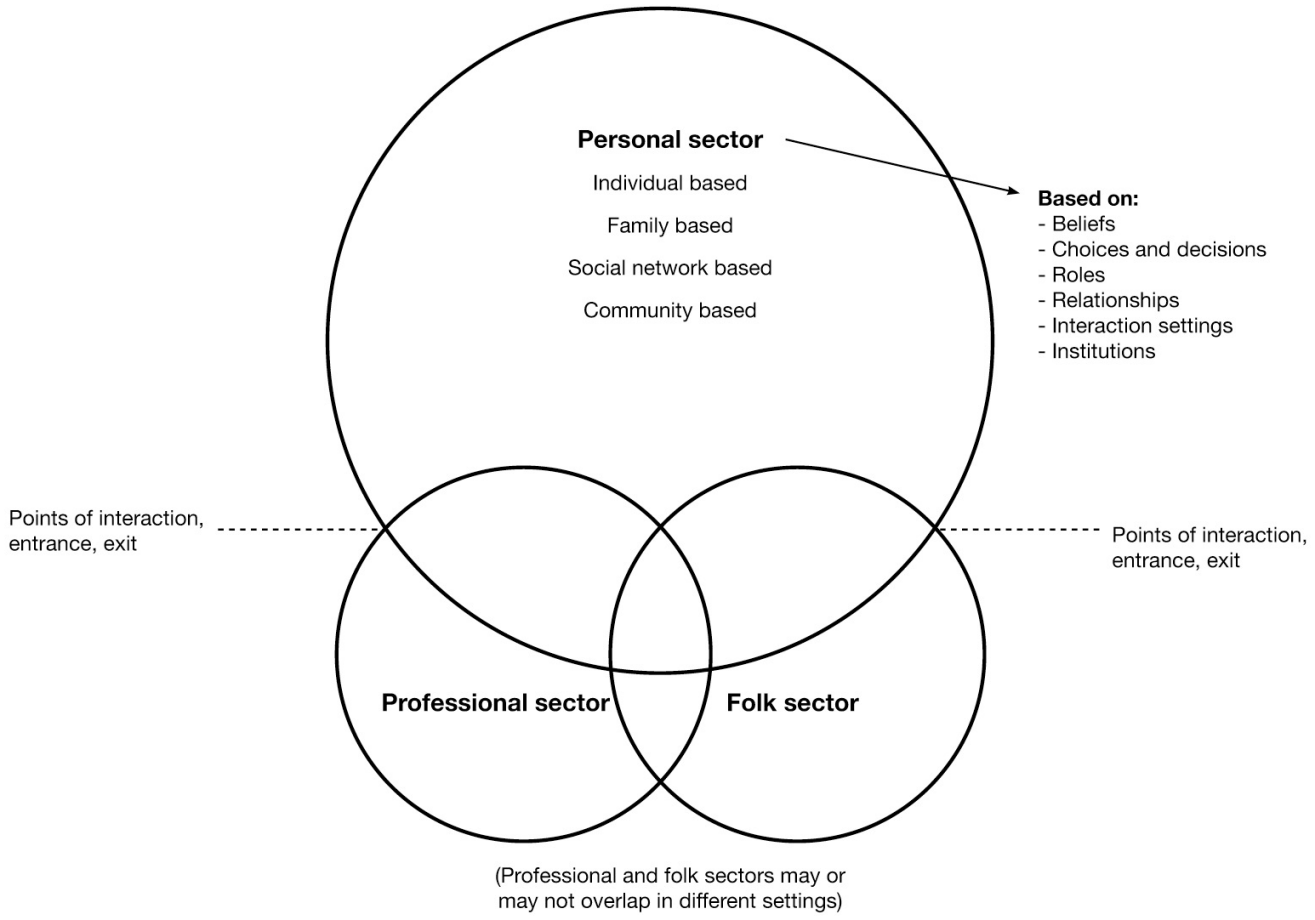
Kleinman’s model of local health care systems, as adapted by Tishelman [25]. Reprinted from [25] under a CC BY license, with permission from Carol Tishelman, original copyright 1993.

After 16 interviews, KB again listened to and re-read all transcripts to inform the final interview. Thereafter, BMB independently listened to all 17 interviews, to evaluate coding and KB and BMB determined jointly that the final interview did not significantly add to or change the substance of the analysis and further recruitment was not necessary. Thereafter, analysis and alternative interpretations were discussed in the full research team. We noted that health care professionals did not appear to play a prominent part either in the strategies or resources for dealing with TSAs described by the participants. Furthermore, most participants did not seem to expect health care professionals to play an active role in managing TSAs. This was in clear contrast to our assumptions of the need for increased engagement of health care professionals, an assumption which also permeates much of the literature. After making an effort to present data in several thematic variations, we found that using the model of local health care systems, developed by medical anthropologist Arthur Kleinman [24], better supported understanding of the findings, highlighting aspects that complement existing literature. It should be noted that this model, presented in Fig 1, did not guide data collection but was applied first in interpreting our findings, to distinguish between the strategies and resources based in participants’ daily lives and their descriptions of health care professionals’ role in TSAs management. Kleinman’s model has served as a theoretical lens to structure and interpret the findings.

#### Kleinman’s model of local health care systems

Kleinman’s model of local health care systems presents a structure for understanding how people respond to sickness experiences in their daily life with or without engaging professionals to manage their condition, rather than an organizational model of society’s provision of health care. The model consists of three, potentially overlapping sectors: the *personal*, the *professional* and the *folk* sectors [24, 25].

*The personal sector*, in the original model referred to as ‘the popular sector’, is the most encompassing sector of the health care system and is composed of lay, non-professional and non-specialist health care. Kleinman [24] argues that majority of efforts at health assessment, maintenance, and treatment take place in the personal sector and may occur on the inter-related levels of the individual, family, social networks, and communities. Signs and symptoms of disease are said to be generally first noticed in the personal sector. The evaluation of an experienced change or a symptom, and the actions then initiated, will be influenced by the cultural context and social relationships around the sick individual, in terms of health beliefs, roles, and relationships. Acting on an experienced change or a symptom may or may not include seeking the expertise of health care specialists; however the choice of whom to consult will to some extent be determined by the settings and institutions available to interact with. Furthermore, consulting a health care specialist implies that the individual enters into one of the other sectors.

*The professional sector* consists of organized health care professionals, whose practice is regulated by society. In the Swedish context, the professional sector is almost exclusively represented by clinicians with a bio-medical perspective. These health care professionals possess social power, as they have the authority to legitimize illness by determining diagnoses and to offer treatment alternatives. Treatment and management of disease is commonly viewed as the responsibility of these health care professionals. Patients, on entering the professional sector from the personal sector, are expected to comply with treatment recommendations. With the diagnosis of a disease, the individual’s role as part of families, social networks and communities in the personal sector may be altered, depending on the course of events in the professional sector and how the cultural context and social networks view the disease.

In the Swedish context, there is very little overlap between the professional sector and *the folk sector* [26], which according to Kleinman is composed of ‘non-professional, non-bureaucratic’ health care specialists, generally without a biomedical background as prerequisite. Traditional examples of specialists in the folk sector are herbalists or bonesetters, however, more relevant examples for this study could be the use of complementary/alternative medicine such as natural products e.g. vitamins, minerals and herbs, or mind and body practices e.g. yoga, meditation and acupuncture [27].

In the model of local health care systems, individuals move back and forth over the intercepts between the different sectors in the health care system and can simultaneously have different roles in different sectors, such as a ‘sick family member’ in the personal sector, a ‘patient’ in the professional sector or a ‘client’ in the folk sector.

## Presentation of findings

The analysis presented here is based on data from four men and 13 women aged 48–77, interviewed between May 2014 and July 2016. To maintain confidentiality, demographic and clinical characteristics are presented on the group level in Table 1. The reported treatments indicate that most participants were in palliative phases of disease, as the Swedish national guidelines for lung cancer treatment recommend chemotherapy, or targeted therapy when applicable, for patients with advanced disease (stages IIIB-IV). Potentially curative treatment with surgery, often followed by adjuvant chemotherapy, is recommended for tumors in stages I-II, and sometimes in stage IIIA [28]. Quotes from participants (in italics) have been selected for illustration. False starts, repeated phrases and irrelevant information have been omitted from quotes, as indicated by /…/ to ease readability without distorting or misrepresenting their meaning. Translation of quotes from Swedish to English was performed by second author (CT), a native English speaker who is fluent in Swedish, and checked by the other authors, all native Swedish speakers fluent in English. Pseudonyms are used in the text.

**Table 1:** Participant characteristics.

		Number of participants (n = 17)
Gender		
	Men	4
	Women	13
Age at time of interview		
	<56	3
	56–65	3
	66–75	8
	>75	3
Time since lung cancer diagnosis		
	3–6 months	9
	6–12 months	5
	1–2 years	2
	>2 years	1
Treatment status		
	Ongoing	10
	Finished/break in treatment	7
Treatment type		
	Chemotherapy	9
	Targeted therapy	6
	Surgery and chemotherapy	2

As noted above, we use Kleinman’s model of local health care systems to structure and present the findings. In this study, all participants were diagnosed with lung cancer, and therefore had established roles as patients in the professional sector. Their sickness had been diagnosed, legitimized and treated, although several mentioned that they knew they would not be cured. When new signs or symptoms emerged, e.g. alterations in their experiences of taste or smell, this was first noticed in the personal sector and also considered in the light of their lung cancer diagnosis and treatment.

**Fig 2:**
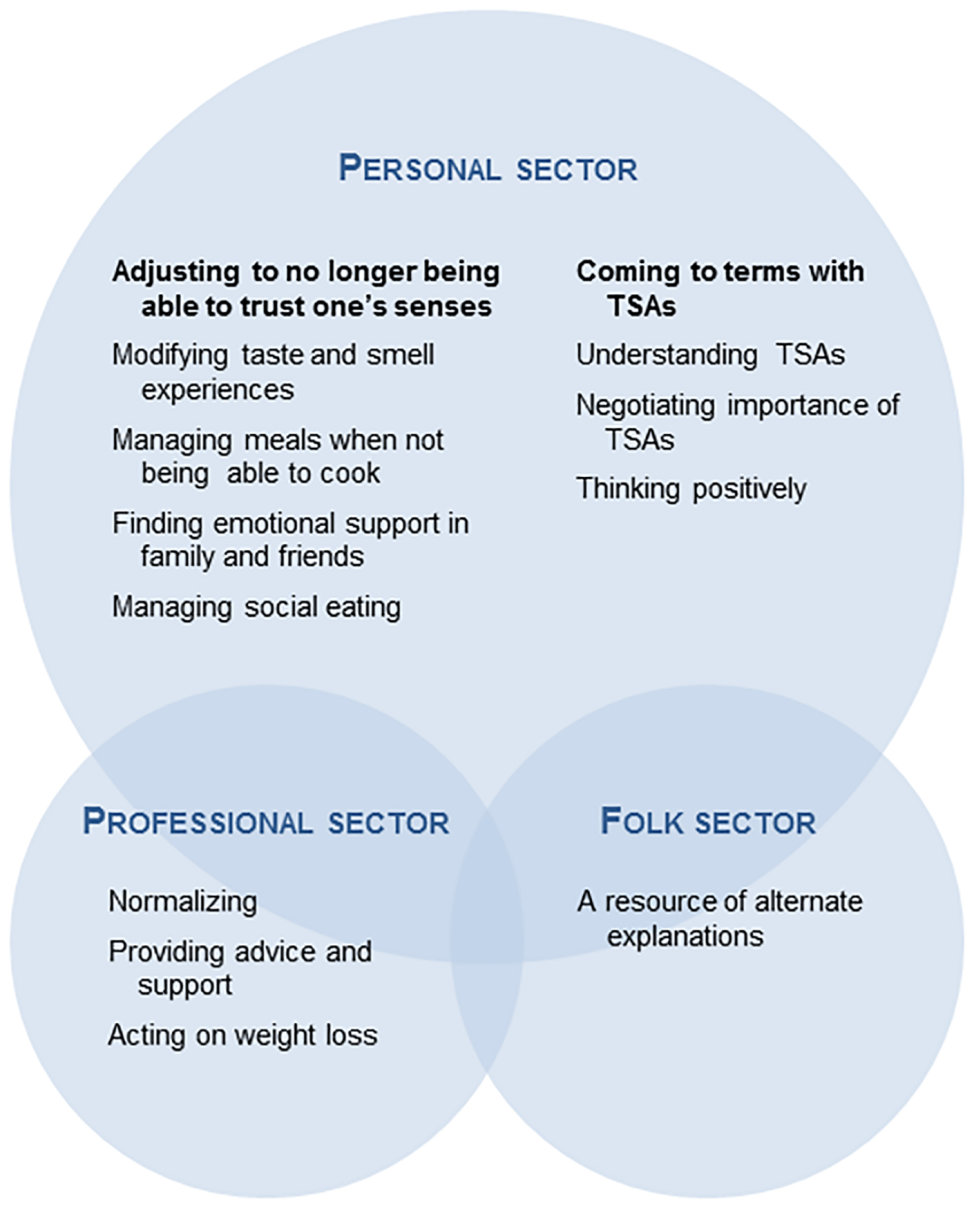
Findings presented in Kleinman’s model of local health care systems. TSAs, taste and smell alterations.

Fig 2 illustrates how the results are structured according to the three sectors of Kleinman’s model of local health care systems. The first section concerns *the personal sector*, where strategies and resources are presented in relation to their context in terms of TSAs characteristics, their consequences and having lung cancer. The second section presents the participants’ perspectives about *the professional sector*, based on their descriptions of their interactions with health care professionals regarding TSAs and related issues. The third section concerns *the folk sector*, based on descriptions from the only participant who reported having used complementary/alternative approaches to manage her TSAs.

### Personal sector

The participants described how TSAs influenced daily life on an individual level, but also in interactions with family, social networks and their communities. Almost all management strategies were undertaken in the personal sector, in participants’ everyday lives. Although participants engaged in various strategies to adjust to TSAs, none of these were said to be able to restore taste or smell function. We found that dealing with TSAs involved two overarching challenges for participants in coping with their daily lives: 1) adjusting to no longer being able to trust information provided by one’s own senses of taste and/or smell, and 2) coming to terms with TSAs, as few, if any, restorative interventions were readily available.

#### Adjusting to no longer being able to trust one’s senses

… *I have lived with that other way of tasting for 64 years or so*…

This quote by Anna, a 65-year old woman, illustrates how participants talked about how they had to adjust to their senses providing different information than they had been used to. Frequent descriptions of taste alterations included increased or decreased sensitivity in general or for specific tastes e.g. salt, sweet, sour or spicy food. All participants described TSAs with regard to specific foods, e.g. coffee, wine or alcohol, red meat and sweets, cakes, or cookies. Several talked about the deceptive nature of TSAs as they are invisible, may come and go unpredictably, and are not perceived by others. Lucas, a 73-year old man, described being at a restaurant with friends and drinking wine he said ‘*tasted like crap’*. He initially felt sure there was something wrong with the wine, but as his companions thought it tasted fine, he came to recognize that his experience was most likely a result of TSAs. Some participants described smell alterations as distinct from taste changes, with increased sensitivity often talked about in terms of specific odors being perceived as bothersome or even offensive, e.g. perfumes, cleaning agents, cooking smells, tobacco smoke, and body odors. Such smells were said to be difficult to avoid in public places. Food or cooking odors from cafés could induce nausea, and a need for fresh air. People smoking, or smelling from smoke, e.g. on public transportation, could be described as unbearable.

Modifying taste and smell experiences: Almost all participants described how they adjusted to TSAs by avoiding those tastes and smells they found unpleasant or even offensive. For many of those with taste changes adjustments included trying out things that might taste better, as taste preferences changed. In addition, some participants reported replacing foods, such substituting tea for coffee, beer for wine, and fish for red meat. These adjustment strategies appeared to be undertaken instinctively, rather than consciously. In contrast, some participants described more proactive strategies, in which they were able to use their knowledge about food and cooking to analyze what worked or did not, and thus consciously try out things that might work better. These participants also often described having a pronounced interest in food and eating-related activities, as knowing how to cook and experiment with different spices and seasonings. For instance, Victoria, a 48-year old woman, described how she had started to reflect upon her taste changes, and how she could adapt: *In part*, *it’s this trying to figure out ‘what can I eat’*? *And thinking about variations on that /…/ trying things out and maybe preparing other stuff than what I’m used to preparing*. *And then with a focus on another kind of ‘taste profile’*, *so to speak*.

Managing meals when not being able to cook: TSAs were also described by some participants as resulting in a loss of interest in or difficulties with food preparation, due to e.g. being bothered by cooking odors or not being able to flavor the food. A few participants, who did not have family members nearby who could help out with cooking, reported having bought ready-made meals when they were unable to cook. These ready-made meals were often described in terms of not tasting good and not being something they would normally eat. Others talked about family and friends as a resource for food preparation. Sometimes others were actively engaged by participants to assist with seasoning when cooking a meal. For example Janet, a 68-year old woman, said:

*Nothing had any taste at all*. *But that’s the way it was for quite a long time*, *and if someone was here to eat*, *then they had to taste the food* (to season it) *you know*, *because I couldn’t taste anything*.

Some also described how family members had taken the initiative to help with cooking, which was many times said to be a much appreciated relief. Compared with other participants’ negative view of having to eat ready-made meals, this appreciation of family members’ assistance with food may have been enhanced by a feeling of being cared for, indicating that such help also was a source of emotional support.

Finding emotional support in family and friends: Further aspects of emotional support described included having a family member or a friend to talk with about TSAs, since TSAs could reduce food enjoyment and evoke feelings of disappointment and frustration. Such emotional consequences were more often referred to by those who described having a previous interest in food and cooking. Others also talked about the importance of being met with respect and understanding for these symptoms. However, participants sometimes spoke about the lack of emotional support. Olivia, a 67-year old woman, spoke about how her husband often forgot she no longer could tolerate the smell of perfumes and cigarette smoke. Despite her requests, he did not quit smoking, and she described with disappointment how she repeatedly reminded him not to use after-shave, to change his clothes, and to brush his teeth after smoking. Although she said that he eventually complied with her requests, she added with a hint of irritation, ‘*It’s no fun to have to repeat it all the time’*.

Managing social eating: Whereas eating with family and friends in most cases was talked about as positive in that it could provide distraction from TSAs, some participants described situations where they experienced an impaired ability to fully take part in social mealtime situations, e.g. toasting with wine, which several participants said they could no longer enjoy. Nicholas, a 74-year old man, described how he could miss having a glass of wine with a good meal on weekends. In addition, he also pointed out how his glass of water became a visible deviation from his established social norm of drinking wine with his family. Eating or dining in more formal situations, e.g. at restaurants or at dinner parties, could imply other challenges. Some participants spoke of their desire to conform to social norms in Sweden, such as eating that which is served and leaving an empty plate, especially when others were unaware of the participant’s TSAs. At the same time, there were often limited possibilities to choose or adjust the taste of the food served in social situations. Therefore, some participants described how they avoided the embarrassment implicit in not being able to finish a meal, e.g. by offering their food to others around the table or, as Patricia, a 76-year old woman, described, taking a only small portion:

*Yeah, well…like, if you’re away, have been out to eat, then it has been really difficult. And then you take…the tiniest amount…first of all, because you have to try it out. Is this also going to taste like vinegar or something else? Then I can’t eat it. And I mean, if you’re invited to someone you can’t just sit there and leave food on your plate, I think. Then they say ‘oh, how little you’re taking’. ‘Yes, but I’ll have more later’. And then people start talking and then they don’t notice. It’s like being an anorexic. You can fool people…into thinking that you’re eating, although you don’t*.

(laughs)

Others said they informed their hosts about their taste problems, and were generally met with understanding. Laura, a 53-year old woman, described having noticed that her hosts had become upset when they realized they had served food she did not tolerate. Therefore, she had learned to tell those inviting her to dinner about her changed taste preferences in advance to prevent similar awkward situations.

#### Coming to terms with TSAs

*My mind was set that ‘yes, this is a side-effect’. It is what it is. Well, one has to accept this… live through it and hope that it will get better in time. I thought that I need to have this medicine… and if it comes along with all these other things, one just has to try to grin and bear it*.

Julia, the 77-year old woman who said the above, was one of several participants describing feeling as if they had no choice but to learn to live with TSAs, as they could not imagine anything that could be done to restore taste and/or smell function.

Understanding TSAs: Although some participants described surprise about their newfound adaptability, one key to coming to terms with TSAs seemed to be an understanding of their cause. Insecurity about what might underlie TSAs could initiate a process of trying to understand their implications based on available information. Nicholas illustrates this when he talks about feeling anxious when first experiencing taste alterations but not knowing they were a treatment side-effect:

*I think you get to be more ‘hypochondria-sensitive’…quite considerably so…and especially in the beginning when you get a diagnosis like this*. *And then you become more observant of yourself and try to figure out what you’re feeling*. *And then something happens so…you have a pain somewhere or something like that…‘Wait a minute*, *is that a metastasis developing*?*’ and so on*. /…/ *I had two metastases right in the beginning*. *In a rib and a shoulder blade* /…/ *But they disappeared and never came back*. *And I had those kinds of thoughts when I began to have taste changes and so on*. *You think about glands and all that*. *That something’s…haywire*.

Knowing his disease had metastasized in combination with being what he refers to as ‘hypochondria-sensitive’, Nicholas’ first interpretation of TSAs was as a possible sign of lung cancer progression. This reasoning resulted in Nicholas feeling anxious rather than constructively contributing to his process of coming to terms with TSAs. In contrast, when talking about his TSAs at the time of the interview, Nicholas said he accepted TSAs *‘with a smile’*, as he found the chemotherapy, which caused the TSAs, to be effective.

Negotiating importance of TSAs: Although participants generally described situations where TSAs interfered with daily life, when asked specifically about how distressing they were, a common response was that they were not so bothersome. This reasoning appeared to be influenced by a process of negotiating various aspects of having lung cancer. Other symptoms, e.g. fatigue or sleeping difficulties, were frequently described as overshadowing TSAs. In addition, the threat imposed by the disease could make participants’ priorities in life more clearly pronounced, for example, in comparison with being unsure they would be alive to see their young children grow up, TSAs were not said to be so important. Other participants, including Julia, quoted in the beginning of this section, talked about how the treatment of her tumor was the highest priority and therefore symptoms and side-effects were subordinate.

Focusing on different aspects of TSAs characteristics or consequences could also support a process of coming to terms with TSAs. Some participants referred to TSAs as acceptable as long as they did not affect food intake enough to cause weight loss. Furthermore, for those participants who had experience of taste changes lasting for a short period of time, such knowledge could also work to motivate them to modulate the importance of TSAs. The negotiating process became evident when Edith, a 64-year old woman, responded to a direct question about how taste changes affected her daily life:

*Yeah*, *well it is sort of…that you…are going to meet someone and*, *like*, *the whole coffee situation*. *You sort of can’t…drink coffee and so*. /…/ *But then I feel—ok*, *but it’s*, *like*, *just a week*. *Then it goes away*. *I know that…that it will go away*. *So it feels like…’eh*, *but it works anyway’ sort of*. *So you just sort of get through it*, *that week*.

Thinking positively: Another aspect described as a resource for coming to terms with TSAs was positive thinking and keeping one’s spirits up. Being optimistic was talked about as important for well-being in general, and even as promoting eating. Helen, a 75-year old woman, talks about the importance of not letting taste changes get the best of her:

*Of course*, *you think about it some* (taste changes) */…/ compared to before when I could stop at a stand and have some ice cream or…go and buy some candy*. *Of course it becomes…’cause candy…* (laughs) *also has its changes in taste*. *So that it…It’s a shame*. *Of course it limits my life*, *it does*. *But…given the whole picture you have to see…see the diagnosis optimistically*, *you can’t burrow yourself into a hole because of taste changes*.

Helen’s comment seems to emphasize the importance of maintaining hope, rather than focusing on seemingly trivial things like not being able to enjoy candy because of TSAs. Lily, a 75-year old woman, who had undergone a potentially curative treatment consisting of surgery with adjuvant chemotherapy, concisely described a process of acceptance, in which she seems to imply that taste changes are a kind of ‘sacrifice’ she was willing to make in order to have overcome a successful treatment:

…*I’m happy that it turned out the way it did. And if I can’t always sense what something tastes like, I’ll just have to put up with it*.

### Professional sector

This section presents participants’ descriptions of their interactions with health care professionals regarding TSAs and related issues. The professional sector is represented by those working in bio-medically oriented health care institutions, which in these data include outpatient clinics, the hospital where participants received treatment, and in some cases palliative home care teams. Most participants talked about health care professionals as having a limited role in the management of TSAs, as illustrated in Olivia’s responses to the interviewer’s questions regarding interaction with health care professionals about TSAs:

**Olivia**: *No*, *I haven’t talked to anyone* (about TSAs) *really*. *It just hasn’t really come up there* (at the outpatient clinic). *I only met the dietitian once in the beginning*. *And then now when she called*, *because I had lost so much weight*. *So I haven’t talked with…what you can do about it*. *I have no idea*.

**Interviewer**: *Is there something you’ve found lacking?*

**Oliva**: *No, because I think you have to test your way forward. Since it’s so individual. So I think that it doesn’t really matter what they say, because it’s about me, anyway. What I need to…try out. And it’s just about testing and testing*.

Although several participants described having received information about TSAs as possible treatment side-effects, or having mentioned experiencing TSAs to the health care professionals who treated them, few participants described having had more extensive discussions with health care professionals about how to adjust to TSAs. However, as with Olivia, most participants seemed to be content with general information which normalized TSAs as part of the disease, and were able to find their own ways to adjust to their changes in sense of taste or smell. Nevertheless, there were some aspects of health care professionals’ actions that appeared to influence participants’ adjustment to and process of coming to terms with TSAs.

#### Normalizing

Several participants reported feeling prepared for TSAs since health care professionals had included this in information about possible treatment side-effects. By doing so, health care professionals seemed to be seen as conveying that TSAs were normal in the context of lung cancer. This normalization could also occur retrospectively, after TSAs began, as was the case for Nicholas: *Yup*, *I’m pretty sure I’ve said that I have taste changes*. *And they’ve nodded and said ‘yeah*, *one gets those’*. (laughs)

#### Providing advice and support

Those who had discussed TSAs with health care professionals had generally spoken with nurses or dietitians, although some reported having talked with a physician at an outpatient clinic or palliative home care team. Some reflected upon the importance of having at least one professional who was easily accessible and knew who they were when they called. One example mentioned was the participant’s ‘contact nurse’. Participants’ had a direct telephone number to their contact nurse who was supposed to be available during office hours. The contact nurse was repeatedly referred to as someone participants had talked to about TSAs or someone they thought they could have spoken to had they felt the need. Thus, knowing there was someone to turn to in case they needed it constituted a form of emotional support. Victoria spoke of appreciating her contact nurse, and in the following quote she refers to her as being no further than a phone call away:

*But regarding the taste changes*, *I actually think the information was…good*. *And it was both in writing and oral*. *And it meant that when they* (taste changes) *came*, *in part I was a little prepared*, *and in part*, *so even if I didn’t remember what I was supposed to do*, *I knew that I could just pick up the phone* (to call the contact nurse), *I remembered there was something about that* (taste changes). *And I think that that was actually the nicest part*, *because all of these side-effects are the kind you can do something about*, *but maybe not a lot*. *But since there was something you could do*, *rinse your mouth*, *and the straw and…at least that means you’re doing something and not just sitting there and suffering /…/ it makes it easier also*, *I think*, *to deal with*. *Even if it doesn’t help right there and then*, *maybe*, *it still feels like ‘yeah but now I’m doing something*. *Now it will probably be better…* (laughs) *in a while*.*’*

Victoria was one of the few participants who said they had received advice on self-care strategies to manage taste changes, e.g. rinsing their mouth with Vichy-water prior to meals, using a straw so that liquids enter the mouth at the back of their tongue, and a variety of food suggestions. She was somewhat unusual in that she found this advice valuable, whereas others said it was readily available without professional help. However, this advice was valuable not so much in terms of being effective for relieving Victoria’s taste changes, but rather as a source of emotional support in that she was provided with something practical to do. Others, including Olivia, quoted previously, acknowledged the challenges for health care professionals in managing side-effects as individual as experiences of TSAs.

#### Acting on weight loss

Several participants described episodes of involuntary weight loss, which led to interaction with health care professionals. It should be noted that weight loss was commonly described as a result of symptoms such as poor appetite, nausea, or swallowing difficulties, rather than due to TSAs alone. Several participants described how health care professionals, in contrast to their response to TSAs, were attentive to and concerned about weight loss or severe eating difficulties that impaired food intake. As Laura explains: *So I just lost weight*, *I think I lost three kilos in one week for a while*. *It just went so fast*. *And that didn’t make them happy*.

Whereas TSAs were often normalized by professionals and their management generally dealt with in the personal sector, participants spoke of situations where it became evident that unintentional weight loss or risk for weight loss was instead a matter of concern for health care professionals. In such situations, concrete actions were said to be initiated, often a referral to a dietitian who provided nutritional advice and treatments aiming at restoring energy and/or protein intake.

### Folk sector

In this study, we did not explicitly ask participants about consultations outside the bio-medical professional sector, however Maria, a 49-year old woman, was the only participant who spontaneously reported using complementary/alternative resources to manage TSAs. In the interview, Maria mentioned having used alternative approaches even prior to her diagnosis, e.g. practicing meditation for several years. She spoke about how ‘normalizing’ information from the professional sector about TSAs as a common side-effect from chemotherapy was not sufficient for her. She continued searching for knowledge and explanations which would support her in her desire to be an active agent in her own symptom management and would ‘compensate’ for what was missing:

*But I still have the same feeling on my tongue. Sometimes it’s… I have… can I show you? There’s some coating now, right? But it’s very pale. It’s pale, isn’t it? Sometimes it’s, after treatment—completely clean. It must mean that everything dies there. I’m very…think it’s quite interesting, because I want to understand. I really want to understand! So I can follow along with my body. So that I can help my body to understand. It’s the worst thing I know, when I don’t understand. And there is no one who can answer*.

Maria found complementary/alternative strategies that better corresponded to her needs, e.g. the practical and measurable activity of adjusting her body’s pH-levels. Maria’s descriptions represent a complex interaction between resources available in all three sectors, in which she negotiated different sources of information in what seems to be an attempt to achieve a sense of control and come to terms with her situation, which for her meant being an active agent in her own care.

## Discussion

In this study we investigate how people with lung cancer and experience from TSAs reason about strategies and resources for dealing with them, and demonstrate how TSAs led to two overarching challenges in daily life. First, the participants engaged in various activities to adjust to their altered sense of taste and/or smell, and at the same time, they underwent a process of coming to terms with TSAs as a feature of having lung cancer. Our findings provide new insight into how the participants primarily dealt with TSAs in the personal sector in their daily lives, with the limited support received for TSAs from health care professionals appearing uncontroversial for participants. Although most participants did not seem to expect health care professionals to play a prominent part in supporting them with TSA management, the professionals’ role was still significant in terms of providing normalizing information, emotional support, some practical advice, and managing involuntary weight loss.

### Strategies, resources and contextual factors

We found that TSAs’ characteristics and consequences informed strategies undertaken by participants to adjust to no longer being able to trust their own senses. In line with results from Bernhardson et al. [17], strategies for avoiding unpleasant tastes or smells were more or less instinctive rather than consciously determined. Such instinctive behaviors were frequently a reaction to experiences of TSAs’ characteristics, e.g. intensity changes in basic taste qualities or smell, or perceiving specific foods or odors to be unpleasant. In contrast, more proactive strategies, e.g. choosing seasonings to adjust the taste of food, relied on participants’ analysis and awareness of the specific characteristics of their TSAs. Thus, in this matter, knowledge and interest in food and cooking constituted an individual resource. Strategies such as avoiding unpleasant odors, adjusting seasonings, and trying out foods are similar to previous research reports’ suggestions for how health care professionals might better support patients with TSAs [29, 30]. As some participants in our study had resources to initiate these strategies by themselves, such advice was sometimes perceived as superfluous. On the other hand, our findings also show that support including food suggestions or strategies to work around TSAs are valuable by offering something practical to do, even if they do not restore taste or smell function per se.

Since friends and family were found to be an important resource, e.g. by helping with cooking and providing emotional support, it should be recognized that there are people who do not have access to a supportive social network. As emotional support was one aspect of adjusting to TSAs, our findings suggest that health care professionals could fill such a gap in resources, in particular by being accessible, willing to talk and aware of practical needs.

Our findings show how participants could come to terms with TSAs by negotiating their importance in relation to TSAs’ characteristics and consequences, the lung cancer diagnosis, and its treatment. Furthermore, personal characteristics, e.g. adaptability or optimism, also seem to facilitate this process by shifting focus towards positive aspects of the situation. However, understanding the cause of TSAs appeared to be of particular importance for the process of coming to terms with them. Thus, the normalizing information about TSAs as a part of the lung cancer treatment, which several participants had received from health care professionals, had potential to promote acceptance of and adjustment to these symptoms. Therefore, offering patients information about TSAs as a side-effect to treatment could be a simple yet important routine through which health care professionals can support their patients in dealing with TSAs.

Although the support provided by health care professionals in terms of normalizing TSAs, emotional support, practical advice, and management of involuntary weight loss, seemed to fulfil most of the interviewed participants’ expectations, it failed to comply with at least one participant’s need to be an active agent in the management of TSAs. Whereas most participants spoke positively about normalization, the information about TSAs being a side-effect of treatment did not meet her needs of understanding the cause of her taste changes in more detail. Instead, she looked for alternative explanations in the folk sector, in complementary/alternative information sources. This exemplifies how both ‘push’ effects from conventional care and ‘pull’ factors to complemenatry/alternative approaches can be in play when people consult complementary/alternative health care specialists [31, 32].

### The role of health care professionals

We found that participants generally initiated management strategies by themselves, which is in line with Hopkinson’s finding about how people with advanced cancer and nutritional problems manage changes in eating habits [33]. Hopkinson sets out to challenge the traditional view of health care professionals as holding the key to solutions for patients’ problems, and discusses how health care professionals could better support them by empowering them to find their own solutions to their eating problems. Given the individual variation in TSAs characteristics, consequences and implications, there is a need for an approach to care that allows health care professionals to identify those patients who need professional support in dealing with TSAs. We are further inspired by Lancely’s discussion about the potential inherent in communication and relationships between health care professionals and patients with cancer [34]. Lancely suggests that the function of health care professionals is to provide support, inspiration and information through listening, perceiving and interpreting the individual’s experiences. Based on this, and the extent of engagement in the personal sector in dealing with TSAs, we argue that TSA support could benefit from an empowering approach to care where definitions of problems and strategies for dealing with them in people’s daily life are shared between patients and health care professionals. One implication would be that health care professionals consider not only individual TSAs characteristics and consequences, but also available resources and the nature of the social situation for the individual. Furthermore, an empowering approach to care might contribute to people feeling part of a joint effort and better identify individual needs of information and support.

In contrast to health care professionals’ rather passive responses to participants’ reports of TSAs, they took a more active approach in relation to risk of insufficient nutritional intake and weight loss. Weight loss and insufficient nutritional intake are measureable problems which fit with the bio-medical focus on bodily symptoms that tend to threaten medical outcomes and have guidelines available for their management [35]. However this focus is dissonant with the subjective, individual, and unpredictable nature of TSAs. In the interviews, participants did not talk about TSAs as a driver of weight loss, except when presenting simultaneously with other symptoms, e.g. nausea or loss of appetite. Our findings therefore complement how previous researchers have discussed TSAs as a primary driver of reduced food intake [3, 14, 36], and point to the importance of accounting for the interaction among symptoms and other contextual factors when studying causes of weight loss and nutritional intake in relation to cancer.

### Methodological considerations

By using Kleinman’s model, we were able to distinguish among the personal, professional, and folk sectors and acknowledge the importance of the personal sector for the management of TSAs in manners not described earlier. Despite most participants being recruited through health care professionals, which may constitute a possible overrepresentation of contacts with the professional sector, with Kleinman’s model we were able to demonstrate how much work that is undertaken in the personal sector without professional support. Furthermore, Kleinman’s model provided a structure which enabled us to present ‘outlier’ descriptions from the participant who had consulted complementary/alternative information sources, which might have been less clear in other thematic structures. However, it should be remembered that Kleinman’s model of local health care systems was developed nearly 40 years ago, and it is unclear how present-day access to internet-based information and virtual contacts should be considered in the different sectors, although this was not often addressed by participants. There may also be an underrepresentation of contact with the folk sector in these data, with only one participant spontaneously reporting using complementary/alternative approaches. As Kleinman’s model did not guide data collection, we did not specifically raise this issue in interviews, but previous international and Swedish research reports that people often do not disclose information about using complementary/alternative approaches [37, 38].

There are some methodological strengths and weaknesses that should be considered. Much of descriptions and consequences provided about TSAs were in line with those reported in previous research with people with varying cancer diagnoses [12, 14, 15, 18], which suggests that our findings may also be relevant for those experiencing TSAs in relation to some other forms of cancer. However, the results are likely to be less relevant in relation to e.g. head-neck cancer, where the cancer itself and its treatment can result in severe TSAs along with other eating difficulties [39, 40]. Another consideration is that while most participants lived in or around one urban area in Sweden, there is variation in professionals and institutions providing care since two centers for lung cancer treatment are represented and three participants resided in and were treated in other cities across the country. There was a predominance of women among the participants, however there is some evidence that TSAs may be more common among women [11, 20]. On the other hand, Bernhardson et al. [12] reason about gender differences in how patients report treatment side-effects, which may also be applicable for our study as eligibility criteria included having reported TSAs. The credibility of the study is enhanced by the contextualization of findings and provision of detailed information about how the qualitative data was obtained [22, 23]. Data collection and analysis were strengthened in that they were undertaken concurrently and therefore could inform each other, based on continuous reflection and discussions within the research team.

### Conclusion

In this qualitative study, we show how people with lung cancer-related TSAs find ways to deal with these symptoms through adjusting to TSAs in their daily life at the same time as they need to come to terms with them. Most management of TSAs was undertaken in the participants’ daily lives, on an individual level and in interaction with family, social networks and communities. Although the support from health care professionals was described as limited, it had potential to influence some aspects of participants’ adjustment to TSAs, as well their process of coming to terms with them.

## Supporting information

S1 File Summary of interview guide. (DOCX)
