# Supplementary material for: Dealing with taste and smell alterations—A qualitative interview study of people treated for lung cancer
Source: PLoS One. 2018 Jan 23;13(1):e0191117. doi: 10.1371/journal.pone.0191117 (PMC5779655; doi:10.1371/journal.pone.0191117)
Supplement: S1 File — (DOCX) [file pone.0191117.s001.docx]

# Summary of interview guide

Interviews were initiated with the following question: *Could you please tell me about any changes you’ve been experiencing with your senses of taste and smell changes at the moment?*

Subsequent questions were based on the individual informant’s responses and covered the following areas:

- Experiences of taste and smell alterations, including characteristics, onset and timing
- Impact of taste and smell alterations on daily life
- How the informant had adjusted practically to taste and smell alterations
- If and how taste and smell alterations were perceived as bothersome
- If and how food choice, cooking, eating, and mealtimes had changed due to taste and smell alterations
- With whom the informant had talked to about taste and smell alterations (e.g. friends, family, health care professionals, or others)
- If and in which ways the informant had received advice or support regarding taste and smell alterations (from e.g. friends, family, health care professionals, or others)

Towards the end of the interview, some demographic and clinical data was obtained (if it had not come up previously during the interview) e.g.: year of birth, timepoint for diagnosis, treatment type(s), start/end of treatment.
